# *Mycobacterium tuberculosis* sulfurtransferase SseA is activated by its neighboring gene product Rv3284

**DOI:** 10.1002/1873-3468.70117

**Published:** 2025-07-16

**Authors:** Giulia Di Napoli, Alex Fissore, Edoardo Salladini, Eleonora Raccuia, Simonetta Oliaro‐Bosso, Alessia Ruggiero, Rita Berisio, Milagros Medina, Adrian Velazquez‐Campoy, Salvatore Adinolfi, Mauro Marengo

**Affiliations:** ^1^ Department of Drug Science and Technology University of Turin Turin Italy; ^2^ Institute of Biostructures and Bioimaging CNR Naples Italy; ^3^ Department of Biochemistry and Molecular and Cellular Biology, School of Sciences, and Institute for Biocomputation and Physics of Complex Systems University of Zaragoza Zaragoza Spain; ^4^ Instituto de Investigación Sanitaria Aragón (IIS Aragón) Zaragoza Spain; ^5^ Centro de Investigación Biomédica en Red en el Área Temática de Enfermedades Hepáticas y Digestivas (CIBERehd) Madrid Spain

**Keywords:** enzyme, *Mycobacterium tuberculosis*, SseA, SufE_*Mtb*_, thiosulfate sulfurtransferase

## Abstract

Tuberculosis remains a critical global health challenge, which underscores the need for new therapeutic targets. A potential drug target is the rhodanese‐like thiosulfate sulfurtransferase SseA, which plays a role in macrophage infection by *Mycobacterium tuberculosis* (*Mtb*) and its resistance to oxidative stress. In our research, we identified a protein (Rv3284), herein referred to as SufE_*Mtb*_, that interacts with SseA and modulates its activity. Sequence analysis and molecular modeling revealed that SufE_*Mtb*_ enhances SseA enzymatic function by binding to its non‐catalytic N‐terminal domain and favoring an activating conformational change in a regulatory loop of SseA. This interaction appears crucial for effective enzyme activity and the maintenance of redox homeostasis in *Mtb*, making the SseA–SufE_*Mtb*_ complex a potential target for new therapies.

## Graphical abstract

Tuberculosis remains a global health challenge and new therapeutic targets are required. Here, we characterized SseA, a sulfurtransferase from *Mycobacterium tuberculosis* involved in macrophage infection, and its interaction with the newly identified protein SufE_*Mtb*_ that activates SseA enzymatic activity. This interaction is crucial for effective enzymatic activity, making the SseA–SufE_*Mtb*_ complex a potential new drug target.

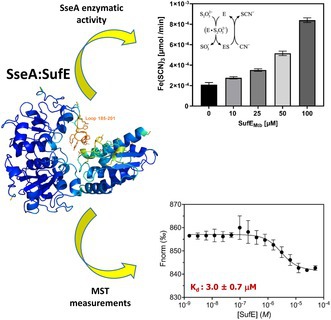

## Abbreviations

**AF**, alphafold

**IMAC**, immobilized metal affinity chromatography

**ITC**, isothermal titration calorimetry

**MDR**, multiple drug‐resistant

**MST**, microscale thermophoresis

***Mtb***, *Mycobacterium tuberculosis*

**SEC**, size‐exclusion chromatography

**TB**, tuberculosis

**TST**, thiosulfate:cyanide sulfurtransferases

**XDR**, extensively drug‐resistant

Tuberculosis (TB) remains a major global health issue and one of the leading causes of death worldwide. The etiologic agent, *Mycobacterium tuberculosis* (*Mtb*), caused 10.8 million cases of TB and 1.25 million deaths in 2023 (WHO). The most urgent problems of TB infections are the capacity of *Mtb* to enter a dormant non‐replicating form through complex modifications of its cell wall [1], which is resistant to drugs and immunoresponse. Also, *Mtb* has an extraordinary ability to develop multidrug‐resistant (MDR) and extensively drug‐resistant (XDR) forms [2]. These events have further emphasized the need to discover new essential functions in pathways different from those targeted by conventional antibiotics to combat this disease [2]. This goal requires more basic research to better understand *Mtb* pathogenesis and immunology, and to identify new targets for diagnostics, drugs, and vaccines [3].

Although its physiological role in *Mtb* has not been clarified yet, the putative thiosulfate sulfurtransferase SseA appears as a potential drug target candidate since it has been demonstrated to be involved in *Mtb* macrophage infection [4] and is overexpressed in MDR and XDR *Mtb* strains [5]. It is suggested to play a fundamental role in the molecular pathways associated with oxidative stress that are necessary for *Mtb* to resist oxidation during infection and for the bacterial cytosolic thiol homeostasis [6]. SseA belongs to a large family of rhodanese‐like enzymes that form a transient sulfane sulfur during catalysis and are able to use a number of substrates, including low‐molecular‐weight thiols [6]. In particular, two types of sulfurtransferases with tandem rhodanese domains have been described. The first group, widely distributed in both prokaryotes and eukaryotes [7, 8, 9], referred to as thiosulfate:cyanide sulfurtransferases (TST), showed *in vitro* specificity for thiosulfate to produce thiocyanide [7], whereas the second group, referred to as 3‐mercaptopyruvate sulfurtransferases (MST) uses 3‐mercaptopyruvate as a sulfur donor to cyanide to obtain pyruvate and thiocyanate as final products [10, 11]. As for SseA, the protein shows the TST characteristic motif CRXGX[R/T] [10].

According to the accepted mechanism [12], TST enzymes facilitate the transfer of sulfur from thiosulfate (donor) to cyanide (thiophilic acceptor) through a double displacement reaction. Initially, TST/rhodanese acquires a sulfane sulfur atom from a donor (e.g., thiosulfate), leading to the creation of a covalent enzyme‐sulfur intermediate (E‐S) characterized by a persulfide bond at the sulfhydryl group of the reactive cysteine in the active site. Subsequently, the persulfide sulfur is transferred from the enzyme to the cyanide, restoring the enzyme to its original form [9, 13, 14].

There is strong evidence that TST and MST are evolutionarily related, as they are able to interact with the same substrate (cyanide, thiosulfate, 3‐mercaptopyruvate), albeit with distinct kinetics and different affinities, and show a striking similarity in amino acid sequences around the active site (66% of sequence homology between TST and MST in rat liver) [15].

Among rhodanese‐like proteins, a thorough structural and enzymatic characterization was carried out on SseA from *Escherichia coli* [16].

Next to the *sseA* gene (Rv3283), along the *Mtb* genome sequence there is a neighboring gene (Rv3284) that encodes for a yet uncharacterized protein. Commonly, proteins encoded by neighboring genes are expressed and work together in a specific metabolic pathway. It was thus tempting to make the working hypothesis that the Rv3284 protein collaborated with SseA in cysteine desulfuration, by providing the sulfur atom during cyanide detoxification. In this study, we report the purification and the biochemical characterization of the rhodanese‐like protein SseA and of its neighbor gene product Rv3284, which showed high homology with *E. coli* SufE and is able to display a significant increase in SseA desulfurase activity. Biophysical characterization and computational analysis were instrumental in proving that SufE‐like protein modulation is mediated by a direct binding to SseA with a 1 : 1 stoichiometry. Molecular modeling provided insights into a plausible activation mechanism of SseA induced by the interaction between the two proteins. Indeed, this interaction mode provides contiguity between the two active sites and prepares SseA for the unlocking of a regulatory loop, which is absent in the homologous *E. coli* protein, that limits substrate access to the catalytic site in SseA.

## Materials and methods

### Protein production

The synthetic gene of SseA from *Mtb* was ordered from IDT, Inc. (Coralville, IA, USA), as a codon optimized sequence for expression in *E. coli* cells and cloned into the pET‐based vector pETM‐20 [17], using NcoI and NotI restriction sites, as a His‐tagged thioredoxin (TrxA) fusion protein comprising a tobacco etch virus (TEV) protease cleavage site.

*Mtb* SufE‐like protein (hereinafter identified as SufE_*Mtb*_) was amplified by PCR from a pET‐DUET plasmid encoding for the protein already available in the laboratory. The PCR products were checked by 1% agarose gel electrophoresis and purified by a QIAquick‐spin PCR purification kit (Qiagen, Hilden, Germany), double‐digested by NcoI and NotI, and ligated into a pET‐24 expression vector, placing SufE under lactose control and in frame with a His‐tag, glutathione S‐transferase (GST) and TEV protease site at the 5′ end. The cloned sequence used in this study was controlled by DNA analysis (BMR Genomics, Padua, Italy).

*E. coli* BL21‐CodonPlus (DE3)‐RP competent cells were transformed using the supplier's protocol (Agilent Technologies, Ketsch, Germany). 100 μL of cells and 2.0 μL of a 1 : 10 dilution of the β‐mercaptoethanol provided with the cells were incubated on ice for 10 min. DNA (10 ng) was added, and cells were incubated for 30 min on ice, heat‐shocked at 42 °C for 20 s, and cooled on ice for 2 min. 900 μL of preheated (37 °C) SOC medium were added to the transformation reaction and incubated at 37 °C for 1 h under gentle shaking. 200 μL of cell suspension were plated on selective media and incubated overnight at 37 °C.

A pre‐inoculum was prepared by picking a single colony from a plate that was inoculated into 20 mL of 2xYT medium supplemented with the appropriate antibiotic (100 mg·L^−1^ ampicillin for SseA, and 30 mg·L^−1^ kanamycin for SufE_*Mtb*_). The culture was incubated overnight at 37 °C with shaking at 200 r.p.m.

SseA and SufE overexpression were obtained by pouring the pre‐inoculum into 2xYT medium supplemented with the appropriate antibiotic and grown at 37 °C until an optical density (OD) at 600 nm of 0.6–0.8 was reached, followed by induction for 3–4 h by the addition of 0.5 mm isopropyl β‐d‐thiogalacto‐pyranoside (IPTG) at 24 °C. The cell pellet was harvested and frozen. Frozen cells were thawed in a lysis buffer (20 mm Tris/HCl buffer, pH 8.0, 150 mm NaCl, 10 mm 2‐mercaptoethanol, 30 mm imidazole, 5% (v/v) glycerol, 10 mg·L^−1^ lysozyme, 1 mm phenylmethylsulfonyl fluoride (PMSF), 4.2 U·mL^−1^ DNase) and subsequently sonicated and centrifuged. The proteins were purified by Ni‐NTA affinity chromatography and eluted with 20 mm Tris/HCl buffer, pH 8.0, in the presence of 150 mm NaCl, 10 mm 2‐mercaptoethanol, 300 mm imidazole. Purified proteins were cleaved from the tag by TEV protease and dialyzed overnight at 4 °C against 20 mm Tris/HCl buffer, pH 8.0, in the presence of 150 mm NaCl and 10 mm 2‐mercaptoethanol. Final purification was achieved by a second step of Ni‐NTA affinity chromatography. When necessary, further purification was carried out by gel filtration chromatography on an ENrich SEC650 column (Bio‐Rad, Milan, Italy). The purity of the recombinant protein was checked by SDS/PAGE after each step of the purification.

Purified proteins were quantified at 280 nm (SseA ε_280_ 71 515 m ^−1^·cm^−1^; SufE ε_280_ 1490 m ^−1^·cm^−1^) and flash‐frozen.

### SDS/PAGE

Protein samples were diluted at a 1 : 1 ratio with denaturing buffer (0.125 m Tris/HCl, pH 6.8, 50% glycerol (v/v), 17 g·L^−1^ SDS, 0.1 g·L^−1^ bromophenol blue) in the presence of 2‐mercaptoethanol and heated at 100 °C for 5 min. Electrophoretic runs were carried out in a MiniProtean Tetra Cell apparatus (Bio‐Rad) on a 12% acrylamide‐bisacrylamide (37.5 : 1 ratio) gel, using a Tris/glycine buffer system at pH 8.3. Gels were stained with GelCode™ Blue Safe Protein Stain (Thermo Fisher Scientific, Milan, Italy).

### Enzymatic assay

SseA thiosulfate sulfurtransferase activity was assessed by a discontinuous method that quantifies the thiocyanate produced by the enzymatic activity and is based on the absorption of the ferric thiocyanate complex at 460 nm (modified from Sorbo [18]).

Briefly, enzymatic activity measurements were performed at 37 °C in the presence of 10 μm SseA, 50 mm KCN, 1 mm DTT, and 50 mm KH_2_PO_4_, pH 8.6. Samples were incubated for 30 min at 37 °C, and the reaction was started by adding 50 mm Na_2_S_2_O_3_. To test the effect of SufE on SseA enzymatic activity, different concentrations of SufE (0, 10, 25, 50, 100 μm) were added during the 30 min incubation time before adding the substrate.

The assay was performed at least in triplicate at a reaction volume of 1 mL and lasted 30 min, and one unit of enzyme was defined as the amount of enzyme that produces 1 μmol thiocyanate per minute. Enzymatic reactions were stopped by adding formaldehyde up to 3.7% (v/v). 150 μL of the reaction mixture was then mixed with 50 μL of 0.245 m Fe(NO_3_)_3_·9 H_2_O (in 15% HNO_3_) that led to the formation of ferric thiocyanate. This latter reaction was carried out for 10 min and centrifuged at 11 000 *g* and the supernatant was analyzed in a microplate absorbance reader (EnSight, Perkin Elmer, Milan, Italy). The amount of ferric thiocyanate was calculated by using a molar extinction coefficient at 460 nm of 6120 m ^−1^·cm^−1^. Enzymatic activity was reported with respect to the total reaction volume (1 mL).

Enzymatic activities at different temperatures were performed by incubating and performing the assay at the reported temperature.

To determine the optimal temperature for activity, measurements were performed at the following temperatures: 4 °C, 16 °C, 25 °C, 37 °C, and 45 °C. This comprehensive temperature range allowed the identification of the temperature at which the highest level of activity was observed, providing insights into the performance of the system under various conditions.

### SseA thermal stability

The thermal unfolding analysis was performed by using a Tycho instrument (NanoTemper Technologies, Munich, Germany). Measurements were carried out in the presence of 20 μm protein, using Tycho standard capillaries. Data were analyzed with the mo affinity analysis Software v2.1.3 (NanoTemper Technologies).

### MST measurements

Binding affinity between *Mtb* SseA and SufE_*Mtb*_ was investigated by MST using the Monolith NT.115 instrument (NanoTemper Technologies), using the Nano red LED at 100% excitation power and the MST power at 40%. About 0.1 nmol of SseA was labeled using the RED‐NHS labeling second‐generation kit (MO‐L011, NanoTemper Technologies) following the procedure recommended by the manufacturer. The dye carries a reactive NHS‐ester group that covalently binds to primary amines (lysine residues). Labeled SseA concentration was ~ 3 μm, with a degree of labeling of ~ 0.5. Binding measurements were carried out at 25 °C in 50 mm sodium phosphate buffer, pH 7.8, containing 0.05% Tween‐20, in the presence of 10 nm SseA and increasing concentrations of SufE_*Mtb*_ (from 1.5 nm to 50 μm). Fitting curves were obtained by the mo affinity analysis v3.0.5 software (NanoTemper Technologies).

### Isothermal titration calorimetry measurements

The calorimetric titrations were performed in a high‐sensitivity automated Auto‐iTC200 microcalorimeter (MicroCal, Malvern‐Panalytical, Malvern, UK). A 10 μm SseA solution in the calorimetric cell was titrated with a 100 μm SufE solution. Previously, the two protein solutions were chemically matched through fresh buffer exchange with PBS, pH 8.0 (phosphate 20 mm, NaCl 150 mm, DTT 2 mm). A series of 2‐μL injections were performed with a spacing of 150 s, stirring speed of 750 r.p.m., and reference power of 10 μcal·s^−1^. The heat evolved after each ligand injection was obtained from the integral of the calorimetric signal and normalized by the moles of protein injected. The heat due to the binding reaction between the inhibitor and the enzyme was estimated as the difference between the observed heat of reaction and the corresponding heat of dilution by including an adjustable term in the fitting routine accounting for the background injection heat. Applying a model considering a single binding site, the interaction parameters were estimated through nonlinear least‐squares fitting analysis: *K*_a_ (*K*_d_ = 1/*K*_a_) association constant; Δ*H*, interaction enthalpy; and *n*, apparent stoichiometry.

### Bioinformatics analyses

Amino acid sequences of known and orthologous rhodanese and rhodanese‐like proteins were aligned using the clustalw method [19]. The selected UniProt IDs with the corresponding species and protein name are described in Table 1.

**Table 1:** Protein name, species and UniProt IDs in Fig. 7.

Name	Species	UniProt ID
Mtu_SseA	*Mycobacterium tubercolosis*	P9WHF6
Mle_THT3	*Mycobacterium leprae*	Q50036
Mth_TST	*Mycolicibacterium thermoresistibile*	E2QRA0
Mtu_CysA2	*Mycobacterium tubercolosis*	P9WHF9
Mle_THTR	*Mycobacterium leprae*	Q50036
Ser_THTR	*Saccharopolyspora erythraea*	P16385
Avi_RhdA	*Azotobacter vinelandii*	P52197
Eco_MST	*Escherichia coli*	P31142
Mtu_THT3	*Mycobacterium tubercolosis*	P9WHF4
Mth_THTR	*Methanothermobacter thermautotrophicus*	O26719
Eco_YnjE	*Escherichia coli*	P78067

### Building Ssea:SufE Interacting models by alphafold

Structural models for the *Mtb* SseA:SufE interaction were produced by using the alphafold multimer colab v2.3.2 (AF2) (activating the Run_relax and Relax_use_gpu options and with a Multimer‐max‐number of cycles of 20) as well as the alphafold 3 (AF3) server [20, 21, 22], by using the corresponding protein sequences (UniProt ID P9WHF7 and P9WGC3, respectively). For comparison of potential structural changes regarding the free proteins, the coordinates of free SseA were taken from both the crystallographic structure with PDB ID 3HZU and from the AlphaFold Database (AFDB) with ID AF‐P9WHF7‐F1, while the structural model for SufE_*Mtb*_ was taken from the AFDB with ID AF‐P9WGC3‐F1 [23]. In addition, SseA and SufE_*Mtb*_ models were also constructed for comparison by using the AF3 server. Models were analyzed using pymol [24].

## Results

### Identification of a putative SufE‐like gene in *Mtb*

**Fig. 1:**
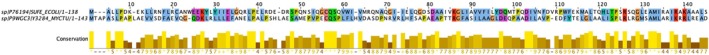
Sequence alignment of *E. coli* SufE and Rv3284 SufE_*Mtb*_ from *M. tuberculosis* (*Mtb*). Color‐code is used to highlight similarities. Glycine and proline residues are colored in orange and yellow, respectively. Other coloring is used to indicate a conserved property according to the following convention: blue, hydrophobic; red, positive; purple, negative; green, hydrophilic. The conservation is highlighted by the yellow bar below the sequences. The amino acid conservation pattern is shown from light (most conserved) to dark yellow (least conserved).

Through a bioinformatics search on the *Mtb* genome, we identified a neighbor sequence to the *sseA* coded as *Mtb* Rv3284. The sequence comparison between *E. coli* SufE and Rv3284 shows 54% similarity (60/112 aa) and 32% identity (34/112 aa), with virtually no insertions/deletions (Fig. 1). *E. coli* SufE is a sulfur transfer protein that promotes a significant increase in the catalytic activity of the cysteine desulfurase enzyme involved in transferring sulfur atoms to synthesize Fe‐S cluster [25]. Different from that observed in *E. coli*, Rv3284 is not part of the Suf operon, but it presents the well‐conserved Cys55 (corresponding to the Cys51 in *E. coli* SufE) essential for SufE to accept *S*_0_ *via* a thiol exchange mechanism [25, 26, 27, 28]. Genomic neighborhood is indeed a reliable indicator for proteins to be involved in the same functional pathways. Consistently, the STRING database predicts the ability of SseA and Rv3284 to form a complex (data not shown). Due to its features, Rv3284 protein is here denominated as SufE_*Mtb*_.

### Purification of SseA and SufE_*Mtb*_

**Fig. 2:**
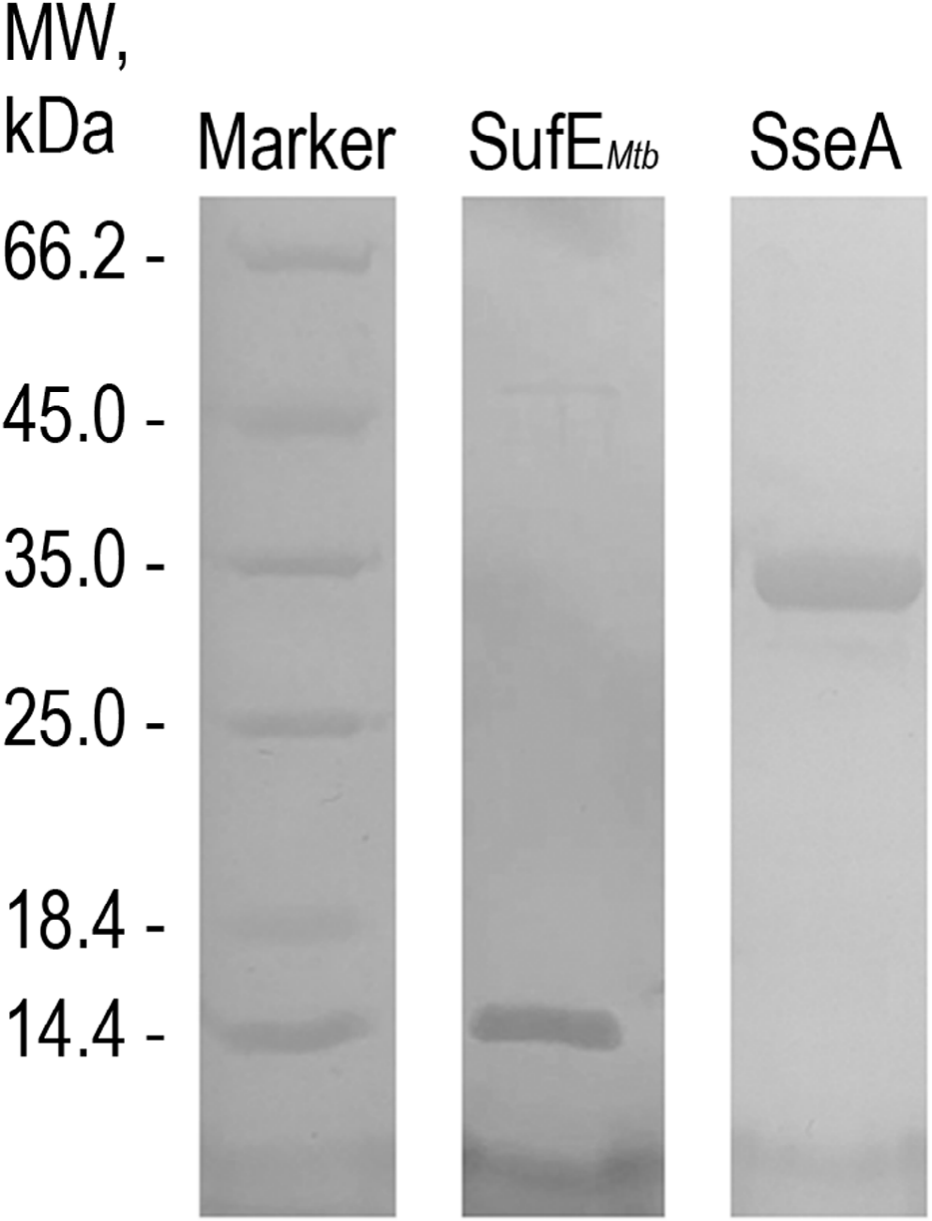
SDS/PAGE of the purified recombinant proteins. SDS/PAGE highlights the high degree of purity of the recombinant proteins purified by immobilized metal affinity chromatography. The band at around 14.4 kDa corresponds to SufE_*Mtb*_ purified to homogeneity, while the band at approximately 35 kDa corresponds to *Mtb* SseA purified to homogeneity. Commercial molecular weight (MW) markers (ThermoFisher Scientific, Milan, Italy) were used as a reference for protein MW.

The setup of convenient purification protocols, leading to the production of soluble isolated highly pure proteins, is fundamental to addressing their individual properties, as well as their interaction properties. The overexpressed SseA and SufE_*Mtb*_ proteins were produced using pETM‐20 and pET‐24 plasmids, respectively. SseA and SufE_*Mtb*_ were purified from the bacterial cell extracts as fusion proteins with His‐tagged thioredoxin and with His‐tagged glutathione‐S‐transferase, respectively, by making use of chromatographic approaches and taking advantage of the inserted six‐histidine tag. The fusion proteins underwent digestion by using the His‐tagged TEV protease, followed by an IMAC chromatography step that allowed obtaining soluble and pure tag‐free SseA and SufE_*Mtb*_, as shown by SDS/PAGE (Fig. 2).

The purified *Mtb* SseA was also analyzed by SEC, highlighting a single peak eluting at a volume compatible with the molecular weight of the monomer (33 kDa), as inferred from the calibration curve (data not shown).

### SseA sulfurtransferase activity

**Fig. 3:**
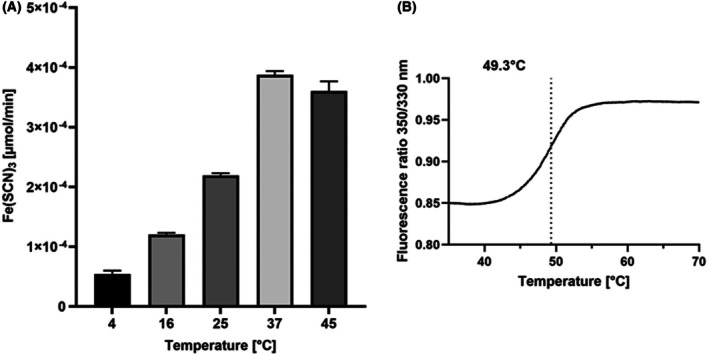
Temperature dependence of *M. tuberculosis* SseA enzymatic activity and protein stability. (A) Bar graph illustrating SseA enzymatic activity (μmol of Fe(SCN)_3_ produced per minute) at various temperature. SseA thiosulfate sulfurtransferase activity was assessed by a discontinuous method that quantitates the thiocyanate produced by the enzymatic activity and is based on the absorption at 460 nm of the ferric thiocyanate complex, as described in the ‘Materials and methods’ section. The amount of ferric thiocyanate was calculated by using a molar extinction coefficient at 460 nm of 6120 m ^−1^·cm^−1^. Data represent the mean ± SD from two independent experiments, each performed in triplicate (*n = *6). (B) Fluorescence measurements (350/330 nm fluorescence ratio) for SseA thermal denaturation. Thermal melting curves were used to determine the melting temperature (*T*_m_), which was found to be 49.3 °C (*n* = 3).

The purified SseA was used for enzymatic activity measurements in the presence of thiosulfate as a substrate, clearly highlighting the ability of the enzyme to transfer sulfur from the substrate to the cyanide acceptor molecule, most probably by the formation of a sulfur‐substituted form of SseA (Fig. 3A). However, sulfur transfer from thiosulfate to cyanide appears moderate, calling for investigating and identifying a more appropriate substrate as a sulfur donor. In this frame, a number of potential acceptors (3‐mercaptopyruvate, glutathione, cysteine) might be used instead of cyanide, which is not physiologically present in cells.

The optimal temperature for SseA enzymatic activity was identified by performing activity measurements over a range of temperatures. Results clearly show that the enzyme exhibits maximum catalytic activity at 37 °C, while a slight decrease is detected at 45 °C. No significant measurements could be performed at higher temperatures, due to protein precipitation (Fig. 3A). These results are consistent with the enzyme melting temperature (*T*_m_) of 49.3 °C (Fig. 3B), as measured by Tycho (NanoTemper Technologies). The decrease in activity observed at temperatures above 37 °C most likely reflects the enzyme approaching its thermal threshold, where structural destabilization begins to occur.

### SseA activity is modulated by SufE_*Mtb*_

A potential effect of SufE_*Mtb*_ on SseA enzymatic activity could be predicted by their gene neighboring location, which appears as a reliable indicator for functional networks of proteins expressed and working together within a specific metabolic pathway. In this context, it has to be highlighted that SufE from *E. coli* promotes a considerable boost in the cysteine desulfurase activity of the iron–sulfur cluster biogenesis Suf machinery, contributes to transfer sulfur to the scaffold for the newly synthesized Fe‐S cluster [25], and shares a high degree of sequence homology with SufE_*Mtb*_.

**Fig. 4:**
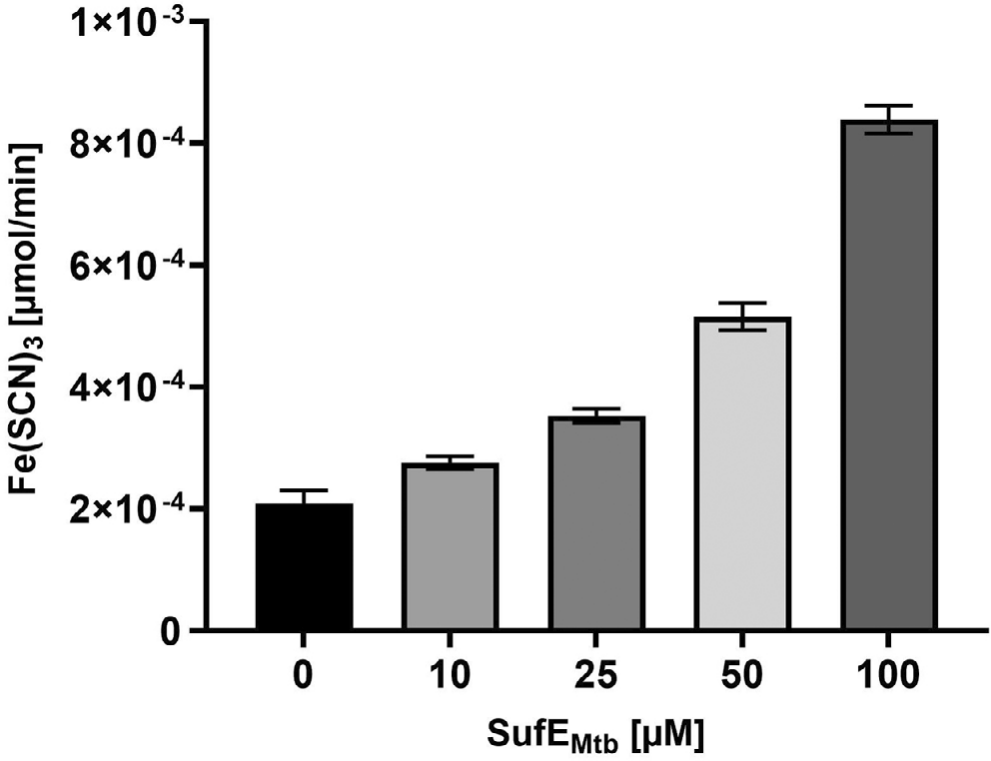
Modulation of thiosulfate:cyanide sulfurtransferase activity of *M. tuberculosis* SseA by SufE_*Mtb*_. The thiosulfate:cyanide sulfurtransferase (TST) activity of 10 μm *Mtb* SseA was assessed—as described in the ‘Materials and methods’ section—in the absence and in the presence of increasing concentrations of SufE_*Mtb*_ at 25 °C. The activity was measured by monitoring the conversion of thiosulfate and cyanide into SCN^−^ and subsequently revealed as Fe(SCN)_3_. Data represent the mean ± SD from two independent experiments, each performed in triplicate (*n* = 6).

To investigate this interaction, SseA enzymatic activity was measured using thiosulfate as a substrate in the presence of increasing concentrations of SufE_*Mtb*_ (10–100 μm). The results revealed that SufE markedly enhances SseA sulfurtransferase activity, achieving a 4‐fold increase at 100 μm SufE_*Mtb*_ (Fig. 4), although it has to be underscored that the measured enzymatic activity rates are substoichiometric, most likely because thiosulfate is not the physiological substrate of *Mtb* SseA.

### SseA and SufE_*Mtb*_ complex formation

**Fig. 5:**
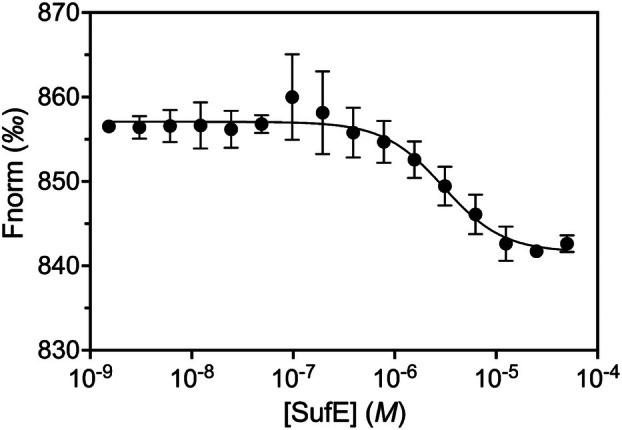
SseA‐SufE_*Mtb*_ interaction *via* microscale thermophoresis (MST). Normalized fluorescence (Fnorm) of labeled *Mtb* SseA when titrated with different amounts of unlabeled SufE_*Mtb*_. Measurements were performed at 25 °C using the NANO red LED at 100% excitation power and the MST power at 40%. The cold region was from −1.0 to 0 s and the hot region from 4.0 to 5.0 s. Fitting curves were obtained by analysis with the mo affinity analysis v3.0.5 software (NanoTemper Technologies). Error bars, SD (*n* = 3).

To address whether the activity increase resulted from a direct protein–protein interaction, MST measurements were carried out in the presence of 10 nm SseA labeled with the RED‐NHS fluorescent dye and increasing concentrations (0.0015–50 μm) of the label‐free SufE_*Mtb*_. Changes in the detected fluorescence signal during MST measurements provided experimental evidence of the protein–protein interaction that appeared relevant to the biological activity of SseA and allowed us to estimate a *K*_d_ value of 3.0 ± 0.7 μm for this interaction (Fig. 5).

**Fig. 6:**
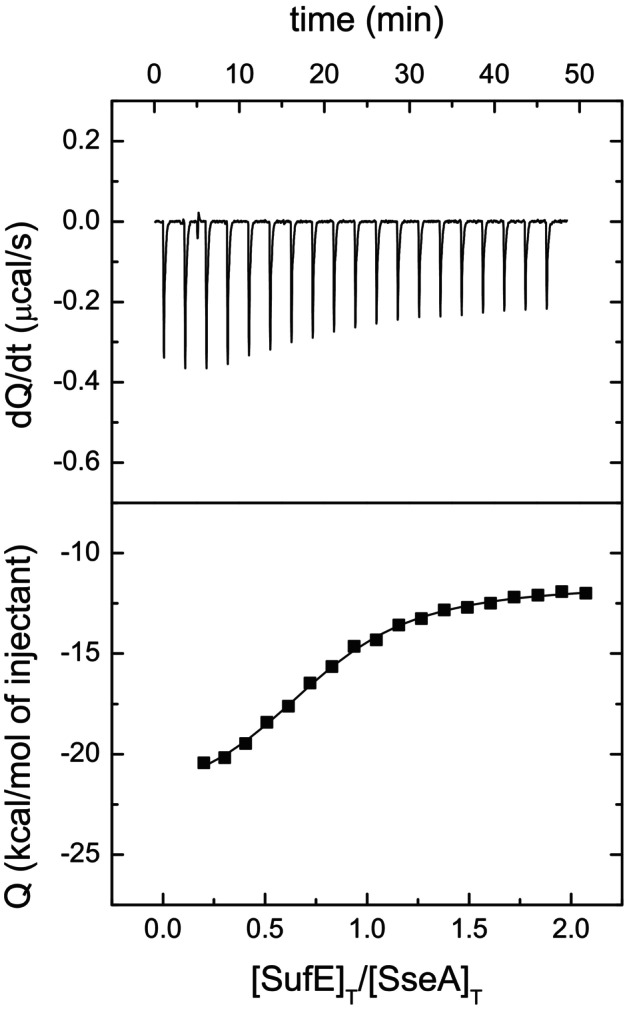
*M. tuberculosis* SseA binding to SufE_*Mtb*_ *via* calorimetric assays. Calorimetric titrations corresponding to the interaction of SseA with SufE_*Mtb*_ in phosphate buffer at 25 °C. Upper panels show the thermograms (thermal power as a function of time) and lower panels show the binding isotherms (titrant‐normalized injection heat effect as a function of the SufE_*Mtb*_/SseA molar ratio within the calorimetric cell). Nonlinear least‐squares fitting (continuous lines in the lower panels) allowed estimation of the interaction parameters: dissociation constant, *K*_d_; observed interaction enthalpy, Δ*H*; and apparent stoichiometry, *n*.

The nature of the interaction between SseA and SufE was also investigated by isothermal titration calorimetry (ITC), which allows measuring binding constants with high accuracy. Titration of SseA with SufE_*Mtb*_ resulted in an exothermic binding, which could fit with a 1 : 1 binding stoichiometry model and provided a *K*_d_ of 3.3 ± 0.6 μm (Fig. 6). These results are in agreement with the data obtained by MST measurements and confirm that the proteins interact with a moderate affinity (low μm range) through an enthalpically driven process (Δ*H* = −11.6 kcal·mol^−1^).

**Fig. 7:**
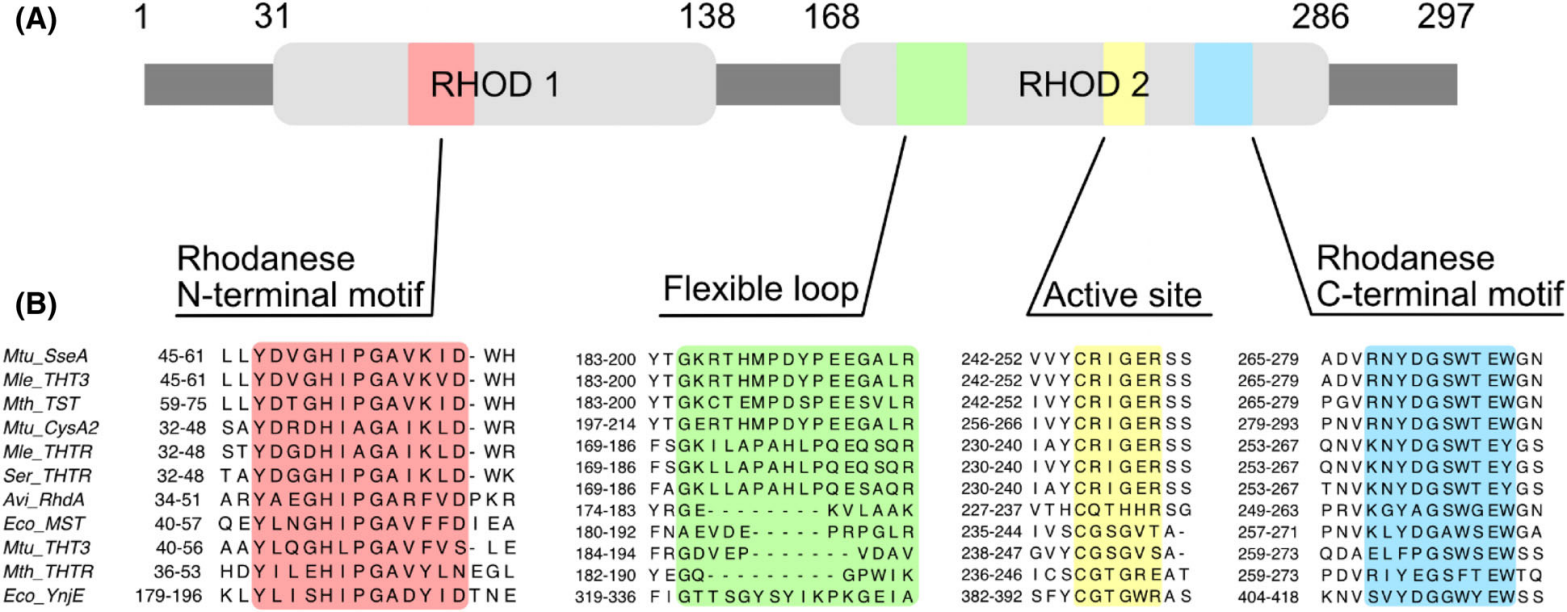
Sequence conservation of relevant regions of *M. tuberculosis* SseA rhodanese domains. (A) Domain architecture of *Mtb* SseA highlighting the presence of two rhodanese domains (Rhod1 and Rhod2). (B) Sequence alignment of selected regions of SseA rhodanese domains with rhodanese‐like proteins orthologues (the selected UniProt IDs with the corresponding species and protein name are described in Table 1). These regions include N (red) and C (blue) terminal motifs of Rhod1 and Rhod2, the active site (yellow) and the flexible loop (green). Numbering in (A) corresponds to the *E. coli* protein.

The binding stoichiometry of 1 : 1 between SufE_*Mtb*_ and SseA, as determined by calorimetric assays, is indeed surprising given the structural complexity of SseA, which contains tandem N‐ and C‐terminal rhodanese domains (Fig. 7A). To address these discrepancies, a sequence alignment of the two rhodanese domains of SseA was carried out in an attempt to elucidate the underlying mechanisms of interaction.

The multiple alignment of rhodanese and rhodanese‐like proteins, focusing on the two rhodanese domains and the active site [10] is shown in Fig. 7. This analysis reveals that, while sequence homology is maintained across the represented domains, certain sequences lack the same disordered loop. Specifically, sequences Avi_RgdA, Eco_MST, Mtu_THT3, and Mth_THTR exhibit gaps in the region highlighted in green. Considering the significance of the observed differences in the interactome of these proteins and the crucial role of the disordered region for the enzyme functionality highlighted in this study, a more detailed investigation into this region will be pursued in future research.

### The molecular Ssea:SufE_*Mtb*_ Interacting complex

**Fig. 8:**
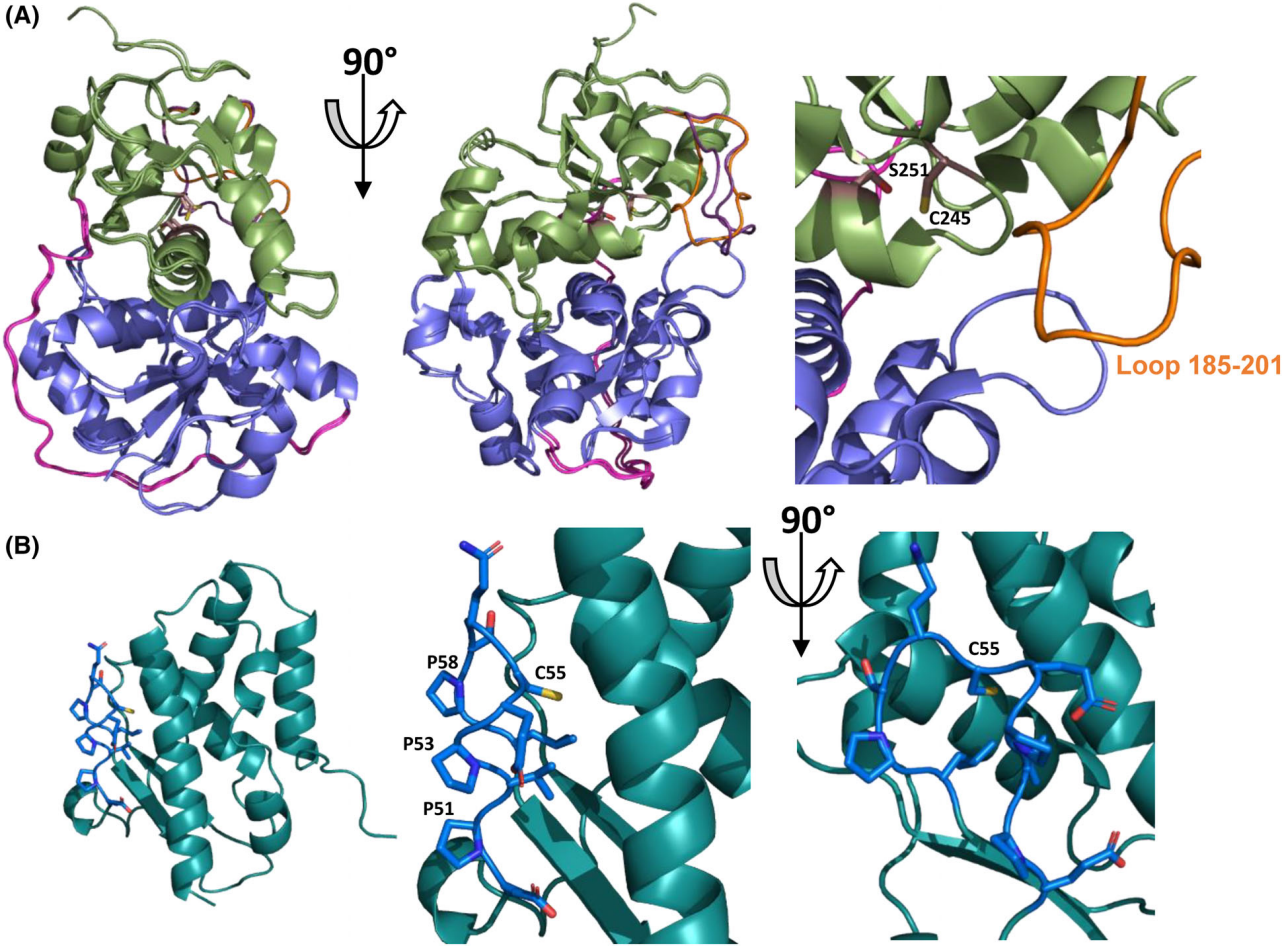
Structural models for SseA and SufE_*Mtb*_ from *M. tuberculosis*. (A) Overlapping of the crystallographic (PDB 3HZU) and AlphaFold (AF‐P9WHF7‐F1) structures for SseA (left panel) and detail of the active site organization (right panel). N‐ and C‐terminal domains are colored in lavender and green, respectively; their connecting loop is in magenta and the loop 185–201 is colored in orange and violet for the crystal and AF structures, respectively. Side chains of Cys245 and Ser251 at the active site are shown in sticks with carbons in brown. (B) Overall AF structural model for SufE_*Mtb*_ (AF‐P9WHF7‐F1) (left panel) and detail of the loop (residues shown in sticks) containing the redox active Cys with its C atoms highlighted in dark blue, while the rest of the protein is in teal.

**Fig. 9:**
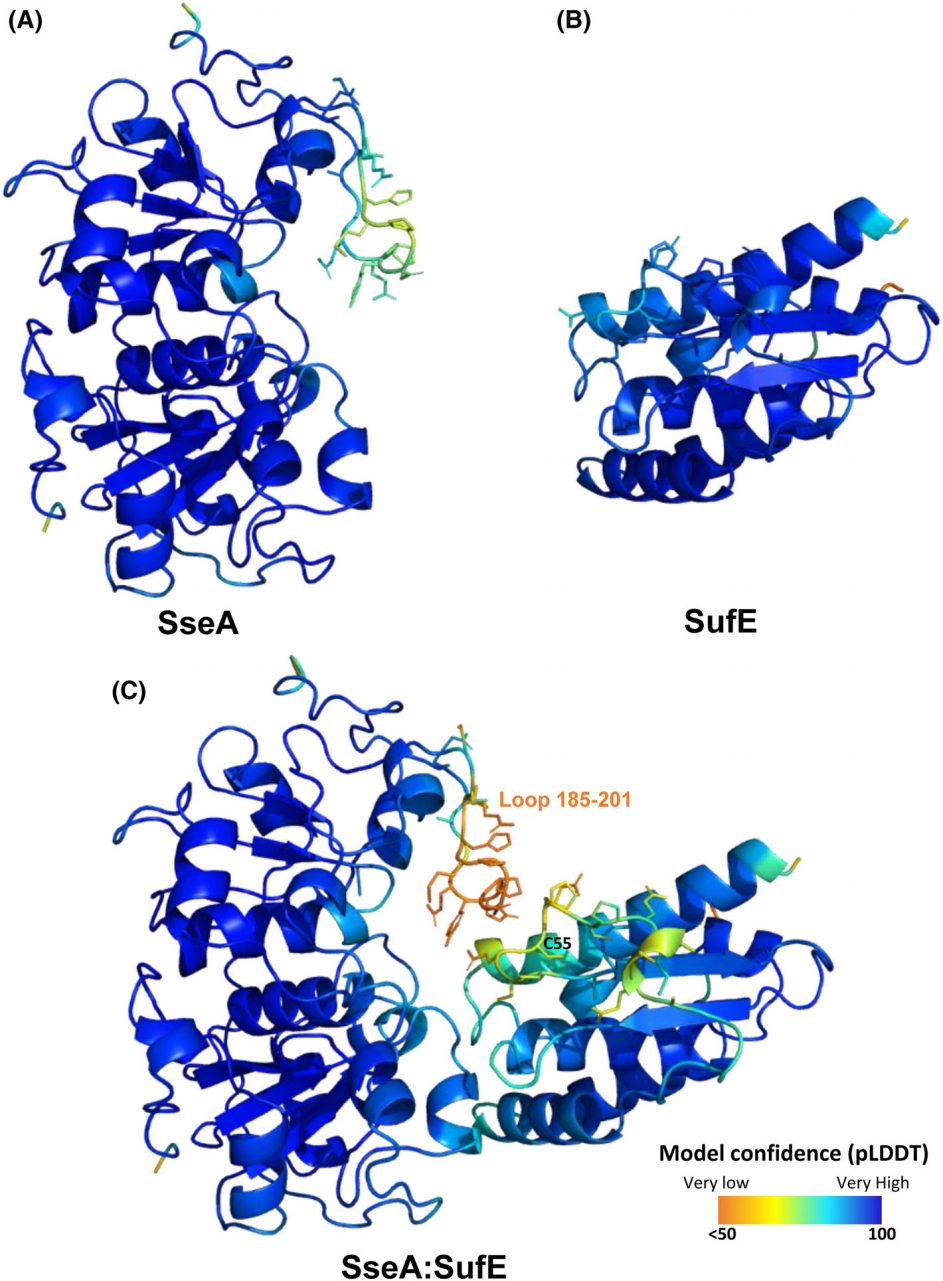
Residue model confidence of free proteins in comparison with the SseA:SufE_*Mtb*_ complex. (A) AlphaFold Protein Structure Database (AFDB) model for *Mtb* SseA (AF‐P9WHF7‐F1). (B) AFDB model for SufE_*Mtb*_ (AF‐P9WHF7‐F1). (C) alphafold 3 (AF3) first model of the SseA:SufE_*Mtb*_ complex colored per‐residue model confidence score (pLDDT) which ranges from 50 (orange) to 100 (dark blue). Side chains of key loops are shown in sticks.

**Fig. 10:**
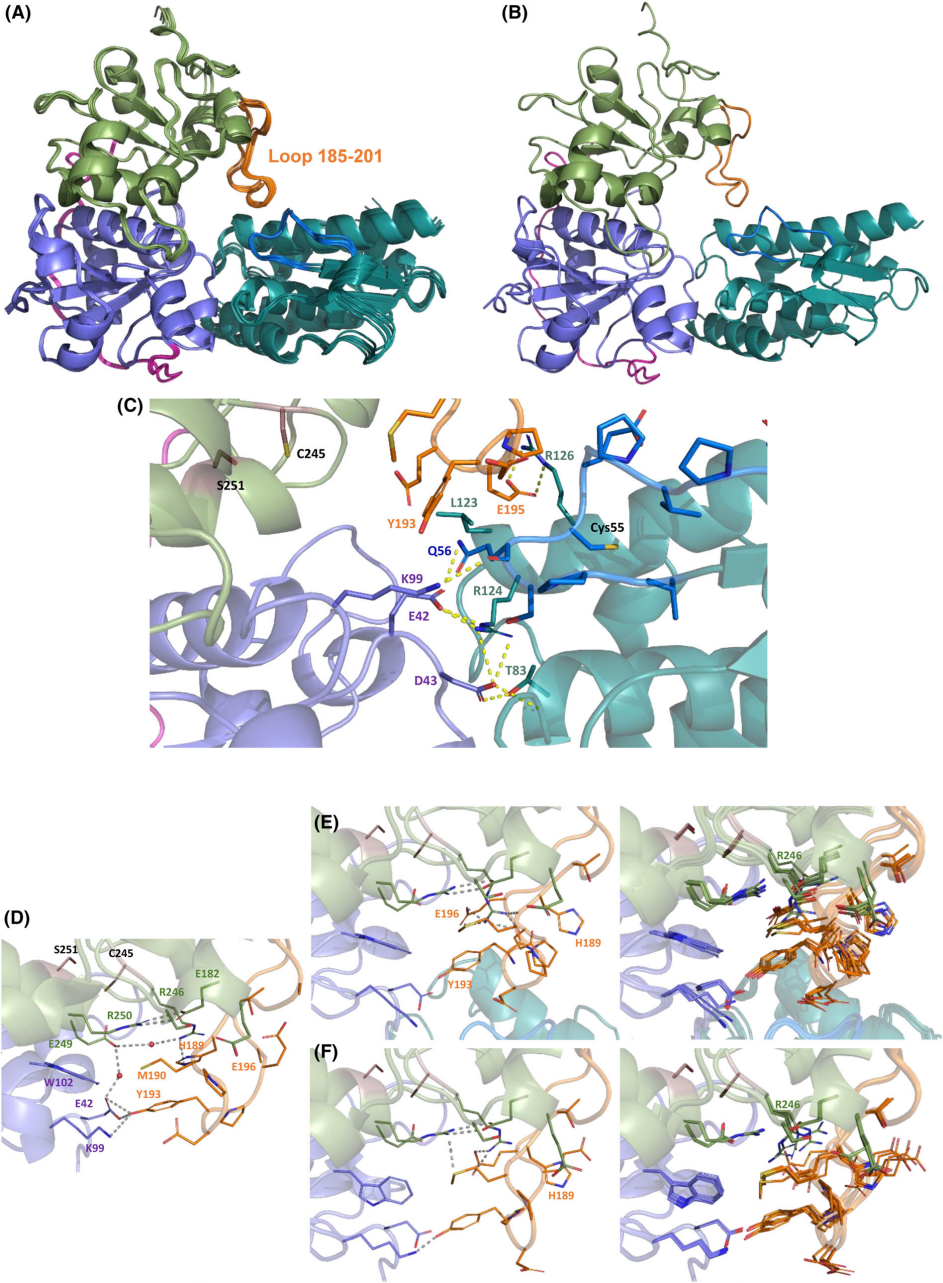
Structural model for the coupling of SseA and SufE_*Mtb*_ from *M. tuberculosis* under non‐reactive conditions. (A) Overlapping of five replicates of the modeling as carried out by alphafold 3 (AF3). (B) Model produced by alphafold 2 (AF2) Multimer Colab. (C) Detail of the interface of the SseA:SufE_*Mtb*_ interaction in the first model provided by AF3. Detail of the conformation of contacts of the 185–201 loop of SseA with its core N‐ and C‐terminal domains in: (D) The *Mtb* SseA crystal structure (PDB 3HZU). (E) The SseA:SufE_*Mtb*_ AF3 model. (F) The SseA AF3 model. In (E) and (F) left panels show the 0 replicate of the 5 produced AF3 models and the right ones the overlapping of the 5 models. N‐ and C‐terminal domains of SseA are respectively colored in lavender and green, their connecting loop is in magenta and the Loop 185–201 is colored in orange. Side chains of Cys245 and Ser251 at the active site are shown in sticks with carbons in brown. SufE_*Mtb*_ is shown in teal, and the loop containing the redox active Cys is highlighted in dark blue. Sidechains of key loops as well as those contributing to polar interactions are shown in sticks, while polar contacts are indicated by yellow (C) and gray (D–F) dashed lines.

*Mtb* SseA features a classical rhodanese‐like fold with N‐terminal (aa 1–132) and C‐terminal (aa 161–296) domains linked by a flexible region (aa 133–161) that wraps around the N‐terminal domain (Fig. 8A) [29]. The RMSD between the PDB and AF models is low (0.5 Å), with notable differences in the 185–201 loop, which respectively exhibit high thermal factors and lower confidence (Fig. 9A). The active site is located within a cleft formed by both domains, with essential catalytic residues Cys245 and Ser251 (Fig. 8A). SufE_*Mtb*_ adopts an αβ compact structure, with Cys55, involved in persulfide formation, situated at the tip of a loop, where its side chain is buried (Fig. 8B). Cys55 has the lowest AF model confidence value (pLDDT 87 for Cα), envisaging mobility during catalysis (Fig. 9B). Generated AF3 and AF2 interaction SseA:SufE_*Mtb*_ models are nearly identical and show high predicted template modeling scores (0.83 and 0.69), indicating a strong confidence in the high quality of the prediction (Fig. 10A,B). SufE engages the N‐terminal domain of SseA, positioning Cys55 at the cleft's entrance (Fig. 10C).

The flexible 185–201 loop at the SseA C‐terminal domain is the only region in contact with SufE_*Mtb*_, which may obstruct access of its reactive Cys55 to the SseA active site. The lower score of the SseA loop in the complex (pLDDT Cα values of 50) compared to the free protein (75) indicates increased flexibility upon interaction (Fig. 9A,C), potentially allowing Cys55 to approach the SseA active site. The SufE_*Mtb*_ loop containing Cys55 also shows reduced confidence, suggesting it may flip to a solvent‐exposed position to facilitate its access to the SseA active site.

The 185–201 loop conformation in SseA differs significantly between AF models and the crystal structure (Fig. 10D–F). In the latest, it interacts with the protein core through several key contacts: Glu42 and Lys99 (N‐terminal domain) H‐bond to Tyr193, while Arg246 (C‐terminal domain) forms a cation‐π interaction with His189 and establishes a water bridge network connecting it to Glu42 *via* Glu249 (Fig. 10D). The AF models show that most core residues remain in fixed positions, with only Arg246 adopting varying conformations due to the twisting of the loop (Fig. 10E,F). Importantly, Arg246 is adjacent to the catalytic Cys245. Conversely, Arg250, located before Ser251, maintains a consistent position, while the interaction between Tyr193 and the C‐terminal domain weakens in the AF3 free SseA model and disappears upon SufE_*Mtb*_ binding (Fig. 10D–F). This envisages that displacement of solvent molecules at the active site or the formation of a sulfur adduct at Cys245 may lead to Arg246 rearrangement, facilitating the 185–201 loop unlocking and promoting SufE_*Mtb*_ complexation.

## Discussion

Tuberculosis (TB) is a critical mycobacterial infection that affects a wide range of mammals, including humans. The main challenges in managing TB are the bacteria's ability to enter a dormant, non‐replicative state that resists drugs and immune response, along with the emergence of MDR strains, which have contributed to the recent rise in TB cases. There is a great need to discover new essential functions in pathways different from those targeted by conventional antibiotics to combat this disease.

In this frame, we characterized SseA, a putative sulfurtransferase, overexpressed in MDR and XDR strains that has been demonstrated to participate in macrophage infection where it may play an essential role in oxidative stress resistance. By bioinformatic analysis, we discovered that the SseA encoding gene presents a neighboring gene, Rv3284. The genomic proximity of this gene, which encodes for a SufE‐like protein, suggested a potential functional relationship, prompting us to investigate Rv3284, herein further referred to as SufE_*Mtb*_.

Alignment of SufE_*Mtb*_ sequence with *E. coli* SufE revealed 60% sequence identity, including the conserved functional residue, Cys55, suggesting functional similarity between the two proteins. Surprisingly, this protein does not seem to be involved in Fe‐S cluster biogenesis as its *E. coli* homolog, but in detoxification of cyanide [30].

To verify this latter hypothesis on SseA role in cyanide detoxification and SufE_*Mtb*_'s ability to regulate it, we purified both SseA and SufE_*Mtb*_ as His‐tagged fusion proteins that allowed for effective isolation. We obtained both proteins in a soluble monomer state and with a high degree of purity.

We proved that SseA is a sulfurtransferase able to transfer sulfur from thiosulfate to cyanide and it shows its maximum activity at 37 °C. Beyond this temperature, we observed a progressive decline in activity mostly mirrored by thermal instability and denaturation of the enzyme.

Having established the role of *Mtb* SseA, we proceeded to assess the role of SufE_*Mtb*_ considering their gene neighborhood, a reliable indicator of proteins working together in a specific metabolic pathway. We proved indeed that SufE_*Mtb*_ promotes a considerable increase in the sulfur transfer activity of SseA. It is important to highlight this is the first time such ability is observed for a SufE‐like homolog, whereas it is well characterized in the Suf operon where SufE is essential to promote SufS activity to carry out Fe‐S cluster biogenesis [31].

Moreover, not all the enzymes of the sulfurtransferase family require an activator and, indeed, this characteristic seems unique to *Mycobacteria*.

To evaluate and further characterize whether the ability of SufE_*Mtb*_ to activate *Mtb* SseA resulted from direct protein–protein interaction, we applied different biophysical approaches that revealed the direct interaction with a moderate affinity (low μm range) and a 1 : 1 binding stoichiometry. It is worth noting that existing literature has already highlighted the functional differences between these domains in terms of sulfotransferase activity [32]. Typically, only the C‐terminal domain presents the catalytic Cys residue necessary for enzymatic function. Multiple sequence alignment analysis reveals that SseA presents a clear sequence homology that is maintained across the represented domains, but certain sequences lack a disordered loop in the C‐terminal domains. Indeed, many representatives of *Mycobacteria* present this extra loop (aa 185–201) that is absent, for example, in *E. coli*. This loop is present mainly in *Mycobacteria* where most likely the sulfurtransferase SseA requires being activated by SufE‐like protein.

We generated models of the SseA:SufE_*Mtb*_ interaction using AF2 and AF3 to investigate the structural basis of their interaction and the potential impact on SseA activation at the molecular level. The two models were virtually identical, suggesting a high level of reliability. Both models indicate that SufE‐like proteins primarily interact with the non‐catalytic N‐terminal domain of SseA. The only contact with the catalytic C‐terminal domain is mediated by a specific loop (residues 185–201).

These structural features could explain the experimentally observed 1 : 1 stoichiometry between SseA and SufE_*Mtb*_ proteins, as the extended loop may sterically or functionally constrain additional interactions.

Moreover, the 185–201 loop in the C‐terminal domain and the region where SufE_*Mtb*_ binds in the N‐terminal domain both appear to hinder access to the active site containing SseA's reactive cysteine. Upon SufE_*Mtb*_ binding, a conformational change has to occur, allowing access to the active site. Notably, both the 185–201 loop and SufE_*Mtb*_ catalytic cysteine region exhibit low‐confidence structural predictions and increased mobility within the complex, a surprising observation, as protein complex formation generally stabilizes each binding partner. These insights suggest that the interaction induces a dynamic structural shift that enables SseA's catalytic function.

Thus, our predictions suggest that the position of the SseA 185–201 loop within the SseA:SufE complex may be highly variable, allowing Cys55 of SufE_*Mtb*_ to approach the SseA active site. Similarly, they suggest that the loop containing Cys55 in SufE_*Mtb*_ can undergo a transition from a buried to a solvent‐exposed conformation, making Cys55 accessible to the active site. Additionally, rearrangements at the entrance of the SseA active site, triggered by the formation of a sulfur adduct, may destabilize the interactions locking the 185–201 loop. This model would suggest an early activation stage in the SseA:SufE interaction, positioning the SufE_*Mtb*_ loop near the catalytic site of SseA and priming it for subsequent catalysis, and should be experimentally validated by altering key interacting residues. A similar behavior has already been reported for *E. coli* SufE that was demonstrated by various studies to activate the cysteine desulfurase SufS not only by acting as the persulfide acceptor, but also by promoting allosteric changes in regions of the SufS structure different from the active site, thus contributing to the enzymatic activity by a dual mechanism [33, 34, 35]. This issue paves the way for further studies aimed at elucidating the actual role of SufE_*Mtb*_ in the sulfur transfer by *Mtb* SseA, taking, however, into account that SufE from *Mtb* and SufE from *E. coli* are, to the best of our knowledge, involved in different sulfur mobilization pathways. In this frame, although this newly identified protein from *Mtb* has been referred to as SufE_*Mtb*_ for consistency with databases (see UniProt ID P9WGC3), naming it SseE (instead of SufE_*Mtb*_) may be more appropriate since it would not imply any possible relationship with the Suf system in that organism [25], thus creating confusion in the literature.

Molecular details and kinetics parameters of SseA enzymatic activity as well as its physiological functions will also be addressed in future work. For this purpose, investigation can focus on several different areas and scenarios since TST are widely recognized to contribute to and participate in a number of processes in cells, ranging from the trafficking of bioavailable sulfur and selenium to the mitochondrial import of 5S rRNA, iron–sulfur cluster restoration, and the degradation of reactive oxygen species [36]. Recently, a central role for *E. coli* SseA has been highlighted in the production of H_2_S that, in bacterial cells, is relevant to redox homeostasis as well as to signaling pathways *via* protein sulfhydration [37]. The understanding of the physiological role of *Mtb* rhodanese‐like SseA may enlarge the functional classification of rhodanese enzymes and eventually help in unraveling the environmental features and the allosteric modulation of its active site.

## Author contributions

SA conceived and supervised the project. AF performed and analyzed the MST and thermal stability measurements. AF and GDN wrote the original draft of the manuscript. GDN and ER purified the proteins and investigated the enzymatic kinetics. ES performed bioinformatic analyses. AV‐C performed and analyzed the ITC measurements. AR and RB performed the cloning and expression of the proteins. MMe performed the computational studies of the interaction between the proteins. SO‐B and MMa helped with the experimental design, contributed to the interpretation of results, and to the final version of the manuscript. All authors provided critical feedback and helped in shaping the research and the manuscript.

## Peer review

The peer review history for this article is available at https://www.webofscience.com/api/gateway/wos/peer‐review/10.1002/1873‐3468.70117.

## Data Availability

The data that support the findings of this study are available in the figures of this article. Raw data are available from the corresponding authors (salvatore.adinolfi@unito.it; mauro.marengo@unito.it) upon reasonable request.
